# Hydrus^®^ Microstent Combined with Cataract Surgery in Pseudoexfoliation Glaucoma Versus Primary Open Angle Glaucoma

**DOI:** 10.3390/jcm15187037

**Published:** 2026-09-11

**Authors:** Leonie F. Keidel, Charlotte Engelmann, Michael Hafner, Miranda Gehrke, Lara Buhl, Siegfried G. Priglinger, Marc J. Mackert

**Affiliations:** Department of Ophthalmology, Ludwig-Maximilians-University Munich, Mathildenstr. 8, 80336 Munich, Germany; charlottejacobi00@gmail.com (C.E.); michael.hafner@med.uni-muenchen.de (M.H.); miranda.gehrke@med.uni-muenchen.de (M.G.); lara.buhl@med.uni-muenchen.de (L.B.); siegfried.priglinger@med.uni-muenchen.de (S.G.P.); marc.mackert@med.uni-muenchen.de (M.J.M.)

**Keywords:** Hydrus, MIGS, PEX glaucoma, primary open angle glaucoma, cataract

## Abstract

**Background/Objectives:** Our objective was to compare clinical outcomes of the Hydrus^®^ Microstent implantation combined with cataract surgery in eyes with pseudoexfoliation glaucoma (PXG) and primary open angle glaucoma (POAG). **Methods:** This retrospective, single-center comparative cohort study included eyes with POAG or PXG undergoing combined cataract surgery and Hydrus^®^ Microstent implantation between June 2021 and December 2025 at the University Eye Hospital of the Ludwig Maximilian University in Munich, Germany. Clinical outcomes were evaluated until 8–14 months after surgery. **Results**: A total of 74 eyes were included, comprising 49 POAG eyes and 25 PXG eyes. After cataract surgery plus Hydrus^®^ Microstent, IOP decreased significantly in both groups from baseline to the final 8–14-month follow-up (POAG: 17.6 ± 3.9 to 13.8 ± 2.3 mmHg, *p* < 0.0001; PXG: 19.2 ± 4.3 to 14.6 ± 2.7 mmHg, *p* < 0.0001). The magnitude of IOP reduction did not differ between groups (*p* = 0.410). Meanwhile, the number of IOP-lowering medications decreased significantly in both groups with no between-group difference (POAG: −1.20 medications; PXG group −1.09 medications, *p* = 0.724). Surgical success rates were comparable. **Conclusions**: Hydrus^®^ Microstent implantation combined with cataract surgery showed comparable surgical outcomes in POAG and PXG eyes. Regardless of the more aggressive disease characteristics of PXG, no significant differences were observed between PXG and POAG eyes regarding IOP reduction, medication burden, BCVA, surgical success, complications or safety outcomes. These findings suggest that combined cataract surgery and Hydrus^®^ Microstent implantation may be a useful treatment option in selected patients with PXG.

## 1. Introduction

Glaucoma continues to be one of the leading causes of irreversible visual impairment worldwide [1]. Elevated intraocular pressure is the most important modifiable risk factor and can lead to progressive optic nerve damage and visual field loss [2]. Primary open angle glaucoma (POAG) is the most common form of glaucoma, whereas pseudoexfoliation glaucoma (PXG) is a secondary open angle glaucoma caused by the accumulation of pseudoexfoliative material in ocular tissues [3,4]. In PXG, this material can accumulate in the anterior segment and trabecular meshwork, leading to impaired aqueous outflow and increased IOP. Compared with POAG, PXG is often associated with higher IOP levels, stronger IOP fluctuations, and a more aggressive clinical course [4].

The first-line treatment usually consists of topical IOP-lowering medication. However, long-term adherence can be difficult, especially in elderly patients, and local side effects may limit the effectiveness of topical therapy [5]. Filtrating procedures such as trabeculectomy or PreserFlo^®^ Microshunt provide substantial pressure reduction, but they are more invasive and therefore are associated with a higher rate of postoperative complications [6,7]. Therefore, less invasive surgical alternatives have been developed for mild to moderate glaucomas, and minimally invasive glaucoma surgery (MIGS) has gained increasing importance in recent years [8,9].

Various MIGS devices are available, differing in their mechanisms of action. The Hydrus^®^ Microstent is an 8-mm Schlemm’s canal-based device designed to improve aqueous humor outflow through the conventional outflow pathway. By dilating and scaffolding Schlemm’s canal, the device may provide access to several collector channels and reduce outflow resistance [10]. When implanted in combination with cataract surgery, the Hydrus^®^ Microstent has shown promising results in lowering IOP and reducing the need for IOP-lowering medication in patients with open angle glaucoma [11,12,13].

Most clinical studies on the Hydrus^®^ Microstent have focused on POAG, while data specifically addressing PXG remain limited. This is clinically relevant because PXG is often more difficult to manage and may present additional surgical challenges, especially in eyes undergoing cataract surgery.

The purpose of the present study was to compare clinical outcomes after Hydrus^®^ Microstent implantation combined with cataract surgery in eyes with PXG and POAG. The main focus was on the evaluation of postoperative IOP, IOP-lowering medication burden, best-corrected visual acuity, surgical success, safety, and the relationship between anterior chamber depth and IOP reduction.

## 2. Materials and Methods

### 2.1. Study Design, Inclusion and Exclusion Criteria

This is a single-center, retrospective comparative cohort study conducted at the Department of Ophthalmology, LMU University Hospital Munich, Germany. Consecutive eyes undergoing combined cataract surgery and Hydrus^®^ Microstent implantation between June 2021 and December 2025 were retrospectively screened. Eligible patients demonstrated ophthalmoscopically detectable glaucomatous optic neuropathy, mild to moderate visual field (VF) loss as defined by Hodappe–Andersone–Parrish criteria, best-corrected visual acuity (BCVA) of 1 or better, Shaffer grade III or IV angle width in all 4 quadrants, and a medicated IOP of 31 mmHg or less. All eyes meeting the predefined inclusion criteria and with sufficient follow-up data were included. Baseline characteristics are summarized in Table 1.

Exclusion criteria were age younger than 18 years at the time of surgery, previous glaucoma surgery in the respective eye, silicone oil tamponade, simultaneous intravitreal injection therapy at the time of surgery, or selective laser trabeculoplasty (SLT) within 90 days before surgery and corneal dystrophy. Both eyes of one patient could be included if both eyes fulfilled the inclusion criteria.

The study adhered to the tenets of the Declaration of Helsinki and was approved by the local ethics committee (Ethics ID: 25-0296). All patients provided written consent prior to any study-related procedure.

### 2.2. Baseline Measurements

Epidemiological data were obtained from each patient, including age, gender, previous ocular comorbidities or procedures and objective refraction-based Snellen chart visual acuity, which was later converted to logMAR for analysis.

Intraocular pressure was measured using Goldmann applanation tonometry. Standard automated perimetry was performed before surgery using a Humphrey Visual Field Analyzer (Carl Zeiss AG, Oberkochen, Germany), and mean deviation (MD) and pattern standard deviation (PSD) values were recorded when available. Anterior chamber depth and lens thickness were obtained from the preoperative biometric assessment.

Postoperative data were collected at predefined retrospective intervals, including day 1, day 2, 1–4 weeks, 4–6 weeks, 3–4 months, 4–6 months, and 8–14 months (mean 12.5 ± 2.4 months for POAG and mean 11.6 ± 2.9 months for PXG) after surgery. All data were extracted from the hospital’s electronic medical record system.

### 2.3. Surgery

The implantation of the Hydrus^®^ Microstent was performed in combination with routine phacoemulsification and intraocular lens implantation under topical or peribulbar anesthesia. All procedures were performed by one experienced glaucoma surgeon at the Department of Ophthalmology, LMU University Hospital Munich, Germany (MM). The Hydrus^®^ Microstent device (Alcon Inc., Fort Worth, TX, USA) is preloaded in a sterile, hand-held delivery cannula. The delivery cannula has a slight curvature to follow the anatomical contour of the anterior chamber angle and Schlemm’s canal.

After completion of cataract surgery, a clear corneal incision was used to introduce the cannula into the anterior chamber. For accurate placement of the device, a surgical gonioprism was used, and the patient’s head position and the surgical microscope were adjusted to allow visualization of the nasal angle. An ophthalmic viscosurgical device was injected to deepen the anterior chamber and widen the angle.

Under direct gonioscopic visualization, the cannula tip was advanced towards the trabecular meshwork and used to access Schlemm’s canal according to the manufacturer’s instructions for use. Using the integrated delivery mechanism, the Hydrus^®^ Microstent was inserted and advanced along approximately 90 degrees of Schlemm’s canal. The proximal part of the stent remained visible in the anterior chamber. After correct positioning was confirmed, the injector was withdrawn and the ophthalmic viscosurgical device was removed.

All surgeries were performed as part of routine clinical care and perioperative management followed the routine clinical standards of the Department of Ophthalmology, LMU University Hospital Munich.

### 2.4. Peri- and Postoperative Management

In both groups, dexagentamicin 5 mL eye drops 4×/d were given for 7 days. In patients who are at risk for developing macular edema nepafenac 1 mg/mL eye drops 3×/d were prescribed additionally for 4 weeks.

Postoperative medication adjustments were guided by the individual target IOP, taking into account pachymetry and glaucoma severity. Ocular hypotensive medications were added when the follow-up IOP exceeded 18 mmHg or when there was evidence of structural or functional disease progression. In patients at increased risk of macular edema (e.g., those with an epiretinal membrane or a history of central retinal vein occlusion), prostaglandin analogues were avoided during the first postoperative month. During this period, patients were treated with either a topical carbonic anhydrase inhibitor or an alpha-adrenergic agonist, alone or in combination with timolol 0.5%. After the first postoperative month, patients requiring continued IOP-lowering therapy were switched to prostaglandin analogues, with additional medications subsequently introduced according to the IOP response.

Surgical success was evaluated at two postoperative intervals: 4–6 months and 8–14 months. The 8–14-month interval was considered the final follow-up endpoint, whereas the 4–6-month interval was analyzed as a secondary mid-term endpoint. Complete success was defined as an IOP reduction of at least 20% from baseline without IOP-lowering medications and without secondary glaucoma surgery, surgical revision, or rescue operation before or at the respective postoperative endpoint. Qualified success was defined as an IOP reduction of at least 20% from baseline irrespective of the use of IOP-lowering medications and without secondary glaucoma surgery, surgical revision, or rescue operation. Medication-free status was analyzed separately and defined as no use of IOP-lowering medications at the respective postoperative time point. Anterior chamber depth and lens thickness were analyzed as additional baseline parameters to evaluate their potential impact on postoperative IOP reduction.

### 2.5. Statistical Analysis

Statistical analyses were performed in R (R Version 4.4.2, R Foundation for Statistical Computing, Vienna, Austria). Continuous variables are reported as mean ± standard deviation and categorical variables as counts and percentages. Because some patients contributed both eyes, analyses were selected to account for within-patient correlation. Baseline age and sex were assessed at the patient level; age was compared using an independent-samples *t*-test and sex using Fisher’s exact test. Eye-level continuous baseline characteristics were compared between POAG and PXG using generalized estimating equations [14] with a Gaussian distribution, exchangeable working correlation structure, and patient-clustered robust standard errors.

Longitudinal changes in IOP, number of topical IOP-lowering medications, and best-corrected visual acuity (BCVA, logMAR) were analyzed using linear mixed-effects models including glaucoma subtype, postoperative time, and their interaction as fixed effects, with age included as a covariate. Random intercepts for patient and eye were included to account for correlation between fellow eyes and repeated measurements within the same eye, respectively. All available observations were included without single-value imputation of missing follow-up measurements. Overall effects of glaucoma subtype, time, and the glaucoma subtype-by-time interaction were assessed using type III F-tests with Satterthwaite approximation of denominator degrees of freedom. Estimated marginal means and corresponding 95% confidence intervals were derived from the fitted models for prespecified between-group and longitudinal contrasts.

Surgical success was evaluated using time-to-event methods. Qualified success was defined as an IOP reduction of at least 20% from the preoperative value, whereas complete success additionally required the absence of topical IOP-lowering medication. To reduce misclassification caused by isolated IOP fluctuations around the success threshold, criterion-based failure was defined as failure to meet the respective success criterion at two consecutive evaluable postoperative visits. The time of the first of the two consecutive unsuccessful visits was assigned as the failure time. Because no subsequent assessment was available after the final 8–14-month visit, failure at this final scheduled interval was accepted without confirmatory follow-up. Eyes without documented failure were censored at their last evaluable visit. As follow-up was recorded in predefined clinical intervals rather than at uniform exact time points, nominal times of 3.5, 5, and 11 months were used for the 3–4-, 4–6-, and 8–14-month visits, respectively. Kaplan–Meier curves were used to estimate the probability of maintained qualified and complete surgical success. Differences between POAG and PXG were assessed using Cox proportional-hazards models with patient-clustered robust standard errors to account for inclusion of fellow eyes; hazard ratios are reported with 95% confidence intervals.

The proportion of eyes free of topical IOP-lowering medication at 4–6 and 8–14 months was analyzed separately as a secondary binary outcome using logistic GEE with patient-level clustering and an exchangeable working correlation structure. As a sensitivity analysis addressing bilateral inclusion, all principal longitudinal and survival analyses were repeated after randomly selecting one eye per patient using a prespecified fixed random seed. In addition, a more conservative survival analysis in which a single unsuccessful visit was sufficient to define failure was performed to assess the robustness of the Kaplan–Meier findings to the failure definition.

As this was a retrospective study of an available clinical cohort, no a priori sample-size calculation was performed. Effect estimates are therefore presented together with 95% confidence intervals, and absence of statistical significance was not interpreted as evidence of equivalence. All statistical tests were two-sided, and a *p* value <0.05 was considered statistically significant.

## 3. Results

### 3.1. Study Population and Baseline Characteristics

The total study cohort comprised 74 eyes from 63 patients, including 49 eyes from 39 patients with POAG and 25 eyes from 24 patients with PXG. Baseline demographic and clinical characteristics are summarized in Table 1. Baseline IOP and the baseline number of IOP-lowering medications were comparable between groups. No statistically significant differences were observed regarding anterior chamber depth, lens thickness, baseline BCVA and PSD. Because postoperative data availability varied between time points, the number of available eyes is reported separately in Table 2. In a sensitivity analysis including one randomly selected eye per patient, the results remained consistent with those of the primary analysis, with no relevant changes in the estimated treatment effect or statistical significance.

### 3.2. Development of IOP

**Figure 1:**
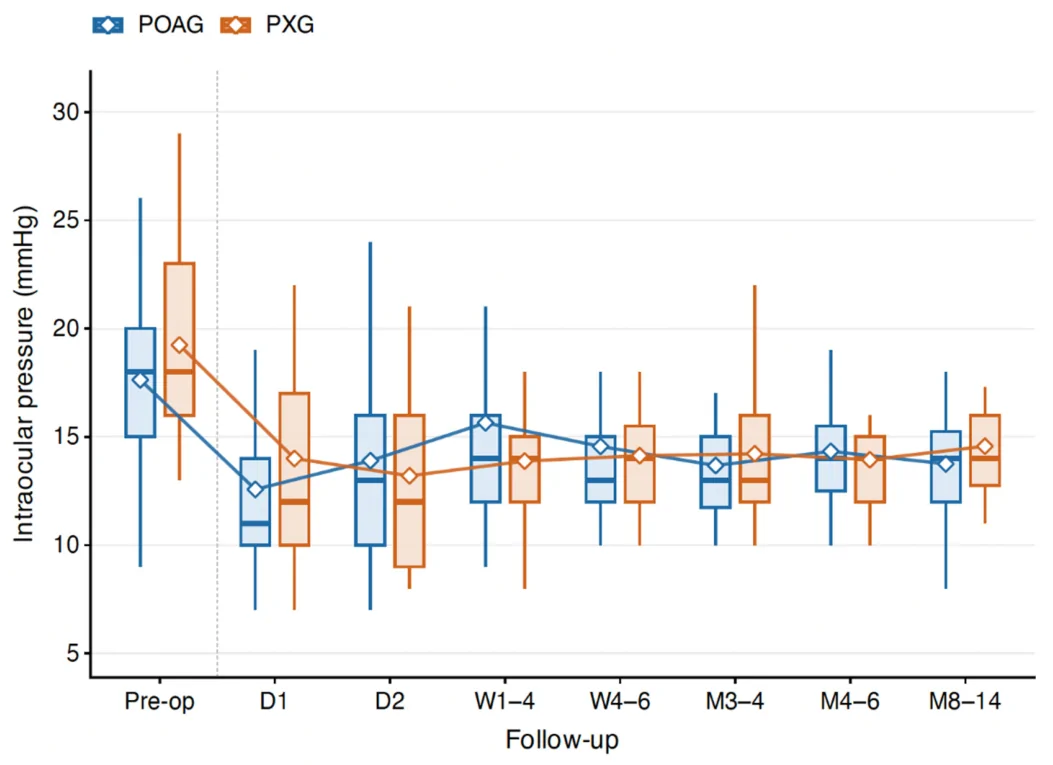
Intraocular pressure. Boxplots of intraocular pressure at each time of follow-up for Hydrus^®^ Microstent implantation combined with cataract surgery in eyes with POAG and PXG. The mean values of intraocular pressure are connected by a line.

Both groups showed a significant reduction in IOP at the final endpoint 8–14 months after surgery: In the POAG group, mean IOP decreased from 17.6 ± 3.9 mmHg at baseline to 13.8 ± 2.3 mmHg at 8–14 months, corresponding to a mean reduction of −4.0 mmHg (*p* < 0.0001). In the PXG group, mean IOP decreased from 19.2 ± 4.3 mmHg to 14.6 ± 2.7 mmHg, corresponding to a mean reduction of −5.1 mmHg (*p* < 0.0001). The magnitude of IOP reduction did not differ significantly between groups at any postoperative time point (*p* = 0.410, Figure 1). Longitudinal analysis showed no statistically significant between-group differences in IOP at all time points (*p* = 0.317 for day 1, *p* = 0.372 for day 2, *p* = 0.056 for 1–4 weeks, *p* = 0.517 for 4–6 weeks, *p* = 0.862 for 3–4 months, *p* = 0.656 for 4–6 months, *p* = 0.863 for 8–14 months).

### 3.3. IOP-Lowering Medical Therapy

**Figure 2:**
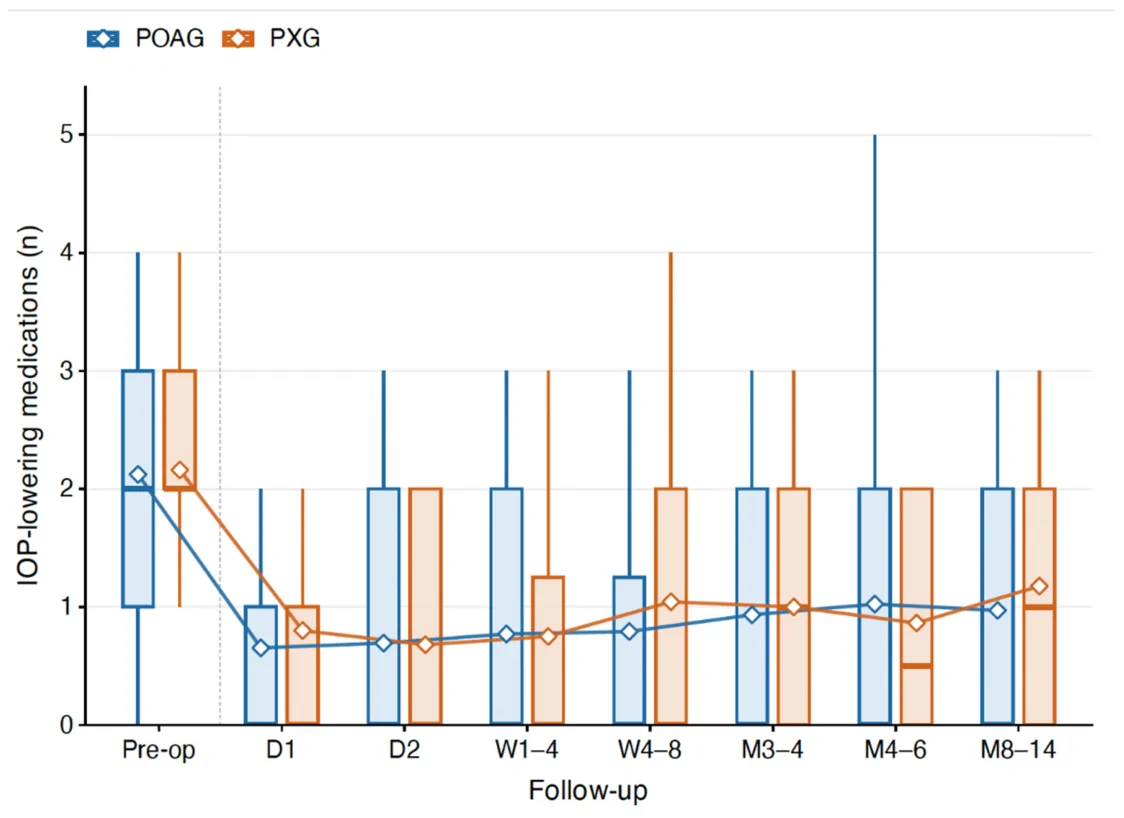
Medication. Boxplots showing the medication at each time of follow-up for Hydrus^®^ Microstent implantation combined with cataract surgery in eyes with POAG and PXG. The mean values of the number of glaucoma medications are connected by a dotted line.

In both groups, a significant reduction in medication could be observed. At the endpoint 8–14 months after surgery, the POAG group required 0.97 ± 1.17 medications (preoperative number of medications: 2.12 ± 0.9, corresponding to a mean reduction of −1.2 medications (*p* < 0.001)); patients in the PXG group required 1.18 ± 1.13 medications (preoperative number of medications: 2.16 ± 1, corresponding to a mean reduction of −1.1 medications (*p* < 0.001)). The magnitude of the reduction in medication did not differ significantly between groups at any postoperative time point (*p* = 0.724, Figure 2). Longitudinal analysis showed no statistically significant between-group differences in medication use at any postoperative time point (*p* = 0.582 for day 1, *p* = 0.978 for day 2, *p* = 0.878 for 1–4 weeks, *p* = 0.428 for 4–6 weeks, *p* = 0.958 for 3–4 months, *p* = 0.569 for 4–6 months, *p* = 0.622 for 8–14 months).

### 3.4. Surgical Success

**Figure 3:**
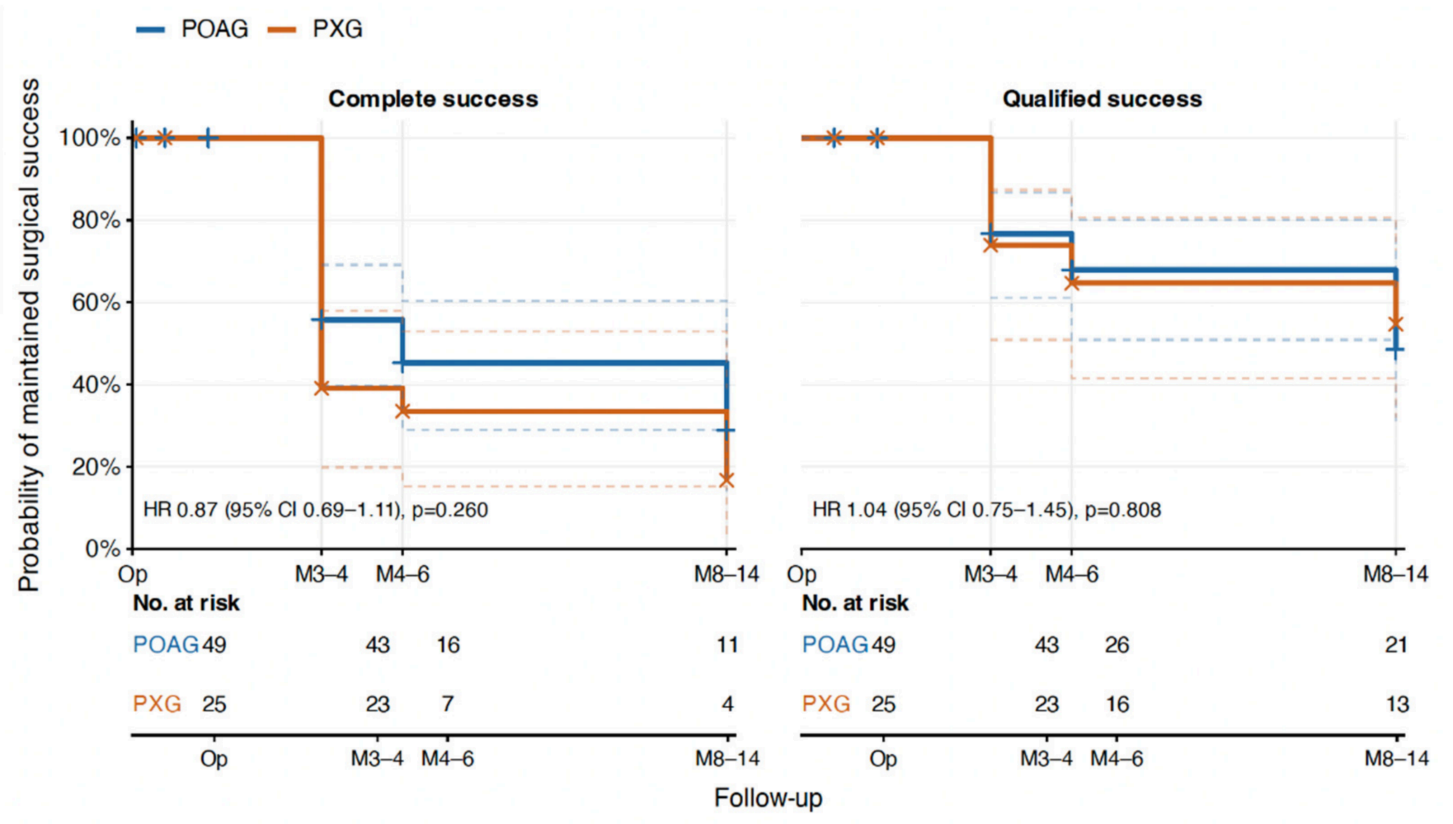
Kaplan–Meier survival analysis of surgical success in eyes with POAG and PXG. Kaplan–Meier curves show the probability of maintaining complete surgical success (left) and qualified surgical success during follow-up. Solid lines represent POAG and PXG, while dashed lines indicate the corresponding 95% confidence intervals. Censored observations are indicated by tick marks. Hazard ratios (HRs) with 95% confidence intervals and log-rank *p*-values are shown within each panel. The numbers of eyes at risk at each follow-up interval are provided below the respective curves. M3–4, months 3–4; M4–6, months 4–6; M8–14, months 8–14.

Kaplan–Meier analysis demonstrated no significant difference in the probability of maintained surgical success between POAG and PXG eyes for either complete or qualified success (Figure 3, Table 3). Although the POAG group showed numerically higher survival probabilities than the PXG group at later follow-up, the difference was not statistically significant (HR 0.87, 95% CI 0.69–1.11; *p* = 0.260). Similarly, for qualified success, the Kaplan–Meier curves were comparable between the two groups, with no significant difference in maintained success over time (HR 1.04, 95% CI 0.75–1.45; *p* = 0.808). See Table 3 for the exact percentages of complete and surgical success for every time point. The percentage reduction in IOP from baseline to the final follow up did not significantly differ between groups (20.4 ± 20% for POAG and 23.6 ± 16.1% for PXG; CI −6.40–15.2, *p* = 0.431).

There was no statistically significant between-group difference regarding the proportion of medication-free eyes at 4–6 months (53.7% vs. 50.0%; 95% CI, 0.638–1.87, *p* = 0.747). Medication-free status remained not significantly different between POAG and PXG eyes at 8–14 months (52.9% vs. 41.2%; 95% CI, 0.706–2.37; *p* = 0.405).

No postoperative complications were documented in the available dataset during the analyzed follow-up period. Close monitoring was performed to allow for an early detection of early IOP elevation, hyphema, stent obstruction or malposition, corneal edema, and intraocular inflammation. No additional glaucoma-related postoperative interventions were required in either group during the analyzed follow-up period.

### 3.5. Development of BCVA

**Figure 4:**
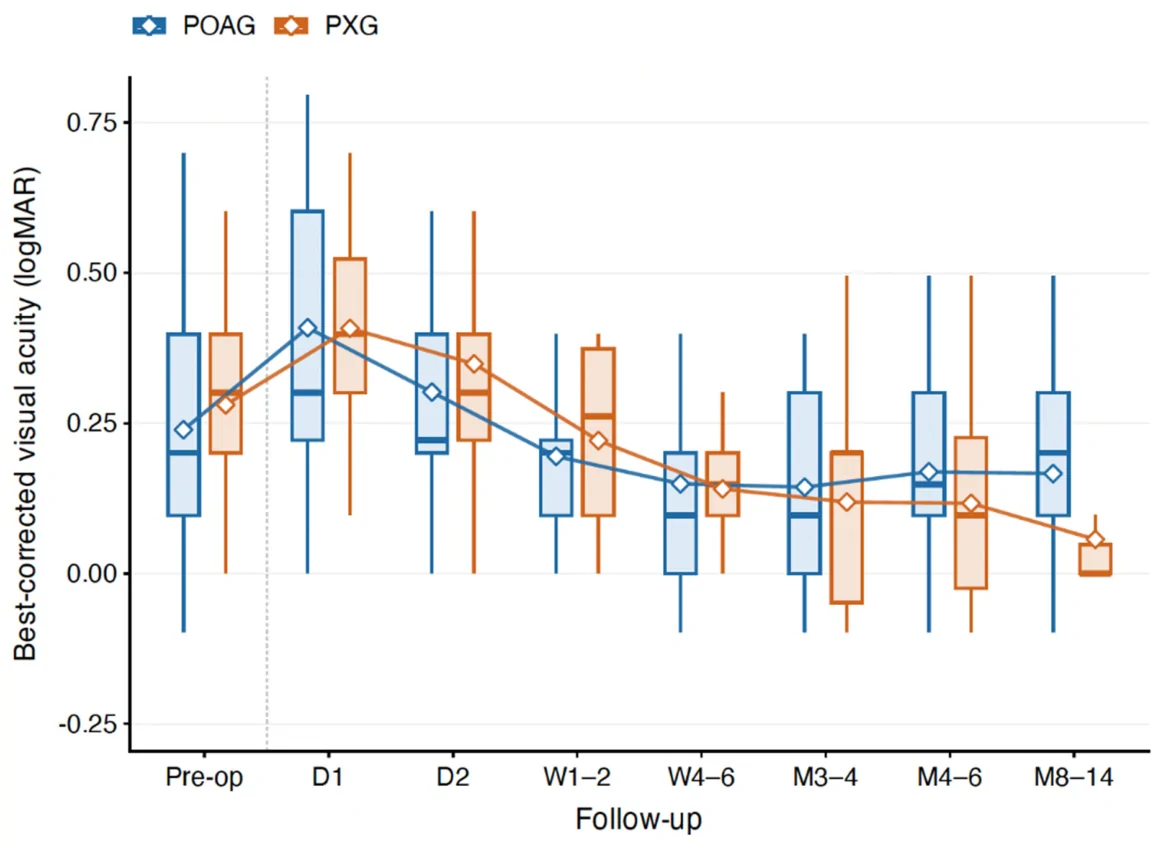
BCVA. Boxplots showing the development of BCVA at each time of follow-up for Hydrus^®^ Microstent implantation combined with cataract surgery in eyes with POAG and PXG. The mean values of BCVA in logMAR are connected by a line.

In both groups, BCVA showed an early postoperative decrease on day 1, followed by gradual improvement during follow-up (Figure 4). At the final 8–14-month follow-up (available BCVA-data for POAG: 21 eyes for PXG 7 eyes), mean BCVA was 0.166 ± 0.159 logMAR in POAG eyes and 0.057 ± 0.160 logMAR in PXG eyes with no significant inter-group difference (*p* = 0.276). Overall, visual acuity improved in both groups after combined cataract surgery and Hydrus^®^ Microstent implantation (*p* < 0.0001 for POAG and *p* < 0.0001 for PXG).

## 4. Discussion

The present retrospective comparative cohort study provides the first real-world evidence on the clinical outcomes of Hydrus^®^ Microstent implantation combined with cataract surgery in eyes with pseudoexfoliation glaucoma compared to primary open angle glaucoma. In the present study, both the POAG and the PXG group showed a significant postoperative reduction in intraocular pressure and in the number of IOP-lowering medications. Importantly, no statistically significant between-group differences were observed in the longitudinal course regarding IOP or medication use. Moreover, surgical success rates, the rate of postoperative complications, and additional glaucoma-related interventions were not significantly different between groups. These results suggest that Hydrus^®^ Microstent implantation combined with cataract surgery may achieve similar outcomes in PXG and POAG eyes.

The development of IOP reported in the present study is in line with previous clinical studies evaluating the Hydrus^®^ Microstent in POAG [13]. In the present cohort, mean IOP decreased significantly from baseline to the final 8–14-month follow-up in both groups. The mean IOP reduction was −4.0 mmHg in POAG eyes and −5.1 mmHg in PXG eyes, with no significant difference in the magnitude of IOP reduction between groups. The magnitude of IOP reduction observed in the present study was slightly lower than that reported in the HORIZON trial for POAG (mean reduction: 8.7 mmHg). However, this difference should be interpreted in light of the substantially lower baseline IOP in our real-world cohort, as no preoperative medication washout was performed. Conversely, the observed IOP reduction is consistent with findings from other real-world studies evaluating implant-based MIGS combined with cataract surgery. In particular, the meta-analysis by Kahale et al. reported a mean IOP reduction of 4.7 mmHg following combined cataract surgery and iStent implantation, which is comparable to the results of the present study.

In the HORIZON study, cataract surgery alone reached similar IOP reduction compared to cataract surgery + MIGS; however, the combination reached better reduction in the number of medications.

That is to say that, in addition to achieving IOP reduction, one of the principal clinical advantages of MIGS procedures is the reduction in glaucoma medication burden. In the present study, the number of IOP-lowering medications decreased significantly in both groups from baseline to 8–14 months, with a mean reduction in drops of −1.2 medications (*p* < 0.001) for POAG and −1.1 medications for PEX. Comparable reductions in medication burden have been reported in previous studies evaluating the Hydrus^®^ Microstent, including both randomized controlled trials and real-world analyses, supporting the role of Hydrus^®^ implantation combined with cataract surgery as an effective strategy to reduce dependence on topical IOP-lowering medication [12,13,15,16]. Notably, the magnitude of medication reduction observed in the present study is consistent with the findings of the HORIZON trial, in which patients undergoing combined Hydrus^®^ implantation and cataract surgery achieved a mean reduction of 1.2 glaucoma medications. This is clinically relevant because reducing topical therapy may improve treatment adherence, decrease ocular surface-related side effects, and reduce the overall burden of chronic glaucoma care, particularly in elderly patients [17,18].

**Figure 5:**
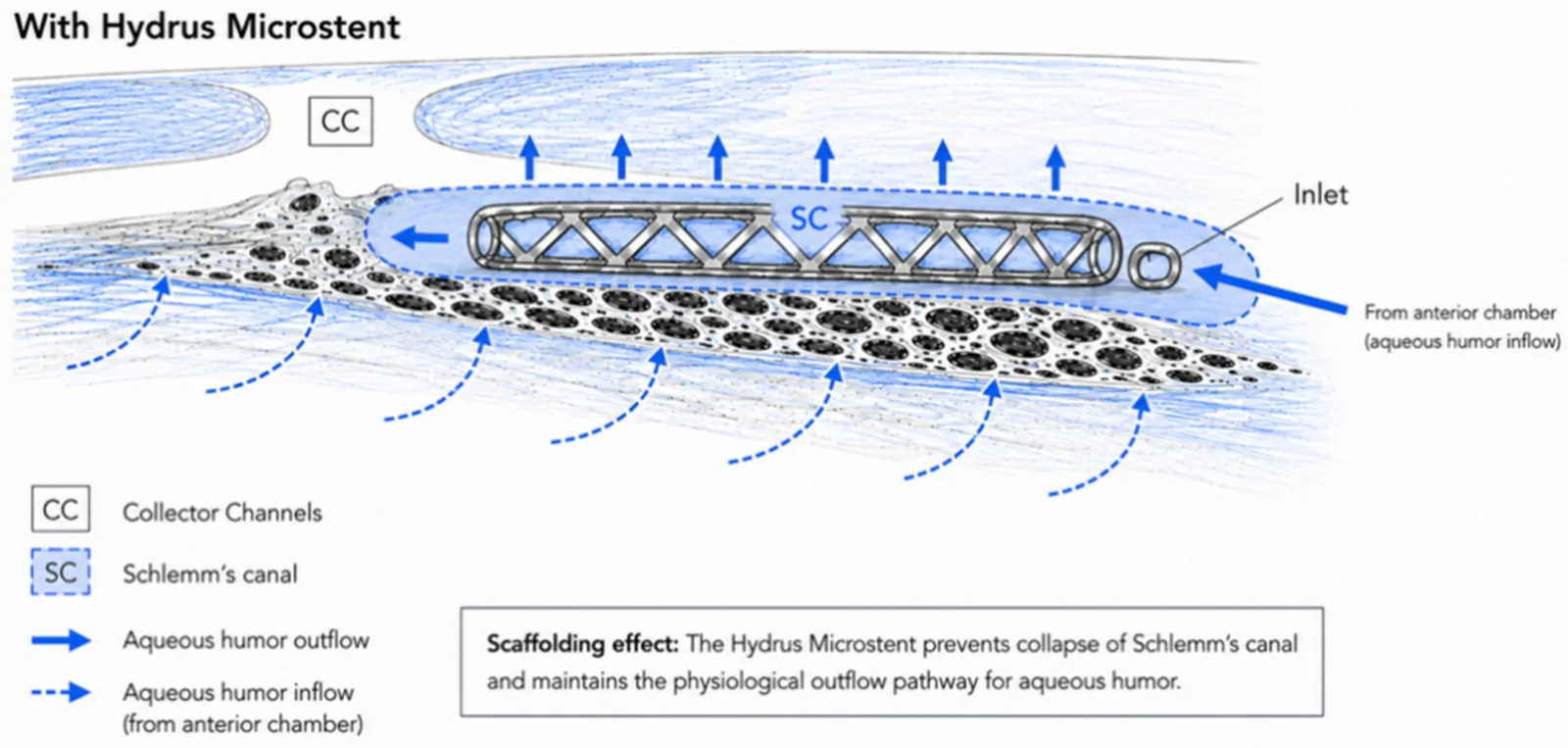
Illustration of the scaffolding and bypass effect of the Hydrus^®^ Microstent in Schlemm’s canal.

The similar reductions in IOP and medication burden observed in POAG and PXG eyes is particularly noteworthy, as PXG is generally regarded as more difficult to treat due to its higher baseline IOP, stronger IOP fluctuations, faster progression, and more pronounced alterations of the conventional aqueous outflow pathway [4]. Despite these characteristics, PXG eyes achieved similar reductions in IOP and medication compared to POAG eyes. In PXG, pseudoexfoliative material accumulates in the anterior segment and especially in the trabecular meshwork (TM) [19]. The pseudoexfoliative material induces profound structural and functional alterations of the TM, including cellular dysfunction with following reduced phagocytic capacity and further accumulation of pigment granules within the outflow system. In addition, PEX is associated with chronic low-grade inflammation: Elevated concentrations of inflammatory cytokines that stimulate abnormal extracellular matrix remodeling and fibrosis within the trabecular meshwork have been identified in the aqueous humor of affected eyes, further increasing outflow resistance [20]. Iris pigment dispersion may additionally lead to obstruction of the trabecular meshwork and may contribute to postoperative intraocular pressure spikes after angle-based surgery. In the present study, despite PEX as a risk factor for complications within MIGS and cataract surgery, no significant difference in effectiveness was observed between POAG and PEX patients. Histopathological findings may provide a mechanistic explanation for the efficacy of the Hydrus^®^ Microstent in PXG. Rasmussen et al. demonstrated by electron microscopy that pseudoexfoliative material can induce focal collapse and splitting of Schlemm’s canal, thereby increasing outflow resistance [18]. Owing to its scaffolding effect, the Hydrus^®^ Microstent may help maintain or restore Schlemm’s canal patency, potentially facilitating aqueous humor outflow despite these pathological changes (Figure 5) [21]. The microstent might furthermore prevent the development of an early rise in IOP after cataract surgery in PEX. Importantly, as in POAG, the pathological alterations in PXG extend beyond the trabecular meshwork and involve the entire conventional aqueous outflow pathway, including the distal collector channels [21]. While trabecular MIGS can improve aqueous outflow at the level of the trabecular meshwork and Schlemm’s canal, persistent dysfunction of the distal outflow system still limits further intraocular pressure reduction in both glaucoma subtypes.

Although cataract extraction likely reduces ongoing iris–lens friction and thus new pigment release, pre-existing pseudoexfoliative fibrillin deposits, chronically elevated aqueous inflammatory mediators, and peristromal fibrosis may still progressively compromise stent patency and distal collector function beyond the 8–14-month window [22]. The present 14-month follow-up provides valuable insights into the mid-term performance of the stent; however, it cannot exclude the possibility of late stent occlusion or a subsequent increase in intraocular pressure, particularly in patients with PEX.

Another important finding of this study was the favorable safety profile during the analyzed follow-up period. No postoperative complications were documented in the available dataset, and no additional glaucoma-related postoperative interventions were required in either group up to the final 8–14-month follow-up. This is relevant because PXG eyes can be more difficult to treat surgically due to pseudoexfoliation-related anterior segment changes (see above). These findings are in line with previous Hydrus^®^ studies, which also reported a favorable safety profile when the Hydrus^®^ Microstent was implanted in combination with cataract surgery [11,12,16]. However, because the present study was retrospective and included a limited number of eyes, the safety results should be interpreted carefully.

This study has several limitations. First, it was a retrospective single-center study, which may limit the generalizability of the results also due to the limited sample size. Second, the PXG group was slightly older than the POAG group, reflecting the epidemiology of pseudoexfoliation syndrome. Importantly, there is no evidence that advanced age affects the IOP-lowering efficacy of trabecular MIGS. Therefore, the age difference between groups is unlikely to have biased the observed outcomes. The third limitation of the present study is the limited availability of BCVA data at the final follow-up visits. Therefore, long-term changes in visual acuity could not be assessed comprehensively. However, BCVA was not a primary endpoint of the study.

Despite these limitations, the present study adds first real-world data on Hydrus^®^ Microstent implantation combined with cataract surgery in PXG. In this cohort, PXG eyes showed comparable clinical outcomes to POAG eyes regarding IOP reduction, medication reduction, BCVA development, surgical success, and documented safety outcomes. These findings suggest that combined cataract surgery and Hydrus^®^ Microstent implantation may be a useful treatment option in selected patients with PXG. Larger prospective studies with longer follow-up are needed to confirm these results.

## 5. Conclusions

In conclusion, this is the first study evaluating the surgical outcome of Hydrus^®^ implantation in combination with cataract surgery in PXG eyes and the first comparison of outcomes with POAG eyes. Both groups demonstrated a significant reduction in IOP and IOP-lowering medication use from baseline to the final 8–14-month follow-up. No statistically significant between-group differences were observed regarding reduction in IOP or medication, development of visual acuity, surgical success, postoperative complications, or additional glaucoma-related interventions. These findings suggest that combined cataract surgery and Hydrus^®^ Microstent implantation may be a useful treatment option in selected patients with PXG. Larger prospective studies with longer follow-up are needed to confirm these results.

## Figures and Tables

**Table 1:** Demographic and clinical characteristics of the POAG and PXG groups.

Variable	POAG (*n* = 49 Eyes)	PXG (*n* = 25 Eyes)	*p*-Value
Age, years, mean ± SD (range)	74.0 ± 6.5 (61.0–84.0)	77.8 ± 5.7 (64.0–87.0)	0.017
Gender (m:f)	13:26	5:19	0.392
Anterior chamber depth, mm, mean ± SD (range)	3.2 ± 0.4 (2.4–4.9)	3.2 ± 0.3 (2.3–3.5)	0.922
Lens thickness, mm, mean ± SD (range)	4.6 ± 0.4 (3.2–5.3)	4.6 ± 0.3 (3.9–5.0)	0.877
Axial length, mean ± SD (mm)	23.9 ± 1.1 (21.6–27.2)	23.7 ± 1.5 (21.6–28.4)	0.758
MD, dB, mean ± SD (range)	−2.6 ± 2.6 (−8.2–1.2)	−4.9 ± 3.0 (−11.3–0.0)	0.028
PSD, dB, mean ± SD (range)	4.2 ± 2.2 (1.7–9.5)	5.8 ± 3.4 (2.2–13.0)	0.107
Baseline IOP, mmHg, mean ± SD (range)	17.6 ± 3.9 (9.0–26.0)	19.2 ± 4.3 (13.0–29.0)	0.135
Baseline number of IOP-lowering medications, mean ± SD (range)	2.12 ± 0.88 (0.00–4.00)	2.16 ± 0.99 (0.00–4.00)	0.954
Baseline BCVA, logMAR, mean ± SD (range)	0.24 ± 0.24 (−0.44–1.00)	0.28 ± 0.22 (−0.10–1.00)	0.404

Values are presented as mean ± standard deviation with range unless otherwise indicated. BCVA = best-corrected visual acuity; IOP = intraocular pressure; MD = mean deviation; POAG = primary open angle glaucoma; PSD = pattern standard deviation; PXG = pseudoexfoliation glaucoma; SD = standard deviation.

**Table 2:** Number of eyes with available data per follow-up time point.

Time Point	Number of Eyes (*n*) POAG/PXG
Baseline	49/25
Day 1	49/25
Day 2	49/25
1–4 weeks	48/25
4–6 weeks	45/23
3–4 months	40/23
4–6 months	35/22
8–14 months	32/20

**Table 3:** Kaplan–Meier analysis of qualified and complete surgical success, showing the estimated probability of maintaining surgical success, 95% confidence intervals, and number of eyes at risk at each follow-up interval.

Outcome	Follow-Up	POAG: Number at Risk	POAG: Survival (%)	PXG: Number at Risk	PXG: Survival (%)
Qualified success	Baseline	49	100.0	25	100.0
	Month 3–4	43	76.7 (95% CI, 61.1–86.7)	23	73.9 (95% CI, 50.9–87.3)
	Month 4–6	26	67.9 (95% CI, 50.9–80.1)	16	64.7 (95% CI, 41.6–80.6)
	Month 8–14	21	48.5 (95% CI, 31.1–63.8)	13	54.7 (95% CI, 32.0–72.7)
Complete success	Baseline	49	100.0	25	100.0
	Month 3–4	43	55.8 (95% CI, 39.9–69.1)	23	39.1 (95% CI, 19.9–58.0)
	Month 4–6	16	45.3 (95% CI, 29.0–60.4)	7	33.5 (95% CI, 15.3–53.0)
	Month 8–14	11	28.9 (95% CI, 14.0–45.6)	4	16.8 (95% CI, 3.4–39.1)

## Data Availability

The raw data supporting the conclusions of this article will be made available by the authors on request.
